# Local strain and tunneling current modulate excitonic luminescence in MoS_2_ monolayers†

**DOI:** 10.1039/d2ra05123k

**Published:** 2022-09-01

**Authors:** Yalan Ma, Romana Alice Kalt, Andreas Stemmer

**Affiliations:** Nanotechnology Group, ETH Zürich Säumerstrasse 4 Rüschlikon 8803 Switzerland mayala@student.ethz.ch astemmer@ethz.ch

## Abstract

The excitonic luminescence of monolayer molybdenum disulfide (MoS_2_) on a gold substrate is studied by scanning tunneling microscopy (STM). STM-induced light emission (STM-LE) from MoS_2_ is assigned to the radiative decay of *A* and *B* excitons. The intensity ratio of *A* and *B* exciton emission is modulated by the tunneling current, since the *A* exciton emission intensity saturates at high tunneling currents. Moreover, the corrugated gold substrate introduces local strain to the monolayer MoS_2_, resulting in significant changes of electronic bandgap and valence band splitting. The modulation rate of strain on *A* exciton energy is estimated as −69 ± 5 meV/%. STM-LE provides a direct link between exciton energy and local strain in monolayer MoS_2_ on a length scale of 10 nm.

## Abstract 2

Local strain in monolayer molybdenum disulfide (MoS_2_) on an evaporated Au surface is studied by scanning tunneling microscopy (STM) induced excitonic luminescence on a length scale of 10 nm.

## Graphical abstract

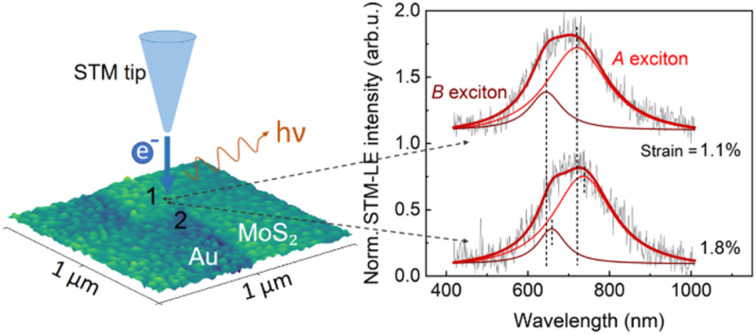

## Introduction

Two-dimensional (2D) monolayer transition metal dichalcogenides (TMDCs), such as molybdenum disulfide (MoS_2_) or tungsten diselenide (WSe_2_), show semiconducting properties with direct electronic band gaps in the near-infrared to visible spectral region.^1–3^ Moreover, monolayer TMDCs possess large exciton binding energies (0.32–0.89 eV),^4–6^ high quantum efficiencies^7,8^ and valley selective circular dichroism.^9,10^ These unique optical properties of monolayer TMDCs are promising for applications in optoelectronic devices, such as light-emitting devices^11–13^ and photon-detectors.^7,14–16^ Theoretical calculations^17,18^ indicated that the electronic band structures of monolayer TMDCs can be tuned by applying mechanical strain. In the following, strain engineering has been reported to modify the optoelectronic responses of TMDCs, not only by controlling the magnitude of the strain^19,20^ but also by controlling the spatial distribution,^21,22^ as proven by photoluminescence (PL) and Raman spectroscopy. However, far-field optical excitation methods are limited in providing information on the nanometer scale due to the diffraction of light.

One promising technique to overcome limitations in spatial resolution is light emission induced by scanning tunneling microscopy (STM). The STM tip works as a low-energy electron source that excites the sample locally. Recently, STM-induced light emission (STM-LE) has been applied to detect excitonic luminescence in monolayer MoSe_2_,^23,24^ tunneling-current-controlled charged and neutral exciton emission in monolayer WSe_2_.^25^ Submolecular resolution in STM-induced electroluminescence has been reported for excitonic and vibronic features of molecules.^26,27^

In this work, we studied the excitonic luminescence of monolayer MoS_2_ by STM-LE. The monolayer MoS_2_ flakes synthesized by chemical vapor deposition (CVD) are transferred onto evaporated gold thin film substrates and excited locally by the STM tunneling electrons. The STM-LE spectra show typical characteristics of radiative decay of *A* and *B* excitons. An intensity saturation of *A* exciton emission is observed when increasing the tunneling current, which can be assigned to exciton–exciton annihilation.^28–30^ Thus, by adjusting the tunneling current one can alter the ratio of *B* exciton to *A* exciton emission.

Moreover, due to the strong van der Waals interactions, the monolayer MoS_2_ conforms to the corrugated Au surface, resulting in locally varying strain in MoS_2_. We observe significant peak (exciton energy) shifts in STM-LE spectra caused by these local strains in MoS_2_. In addition, the valence band splitting is found to be modulated by the strain. We report the first observation of local strain-modulated excitonic luminescence in monolayer MoS_2_ by STM-LE on a length scale of 10 nm. The STM-LE technique offers an efficient approach to studying the optical properties of 2D materials on the nanometer scale.

## Results

### Basic characterization

**Fig. 1:**
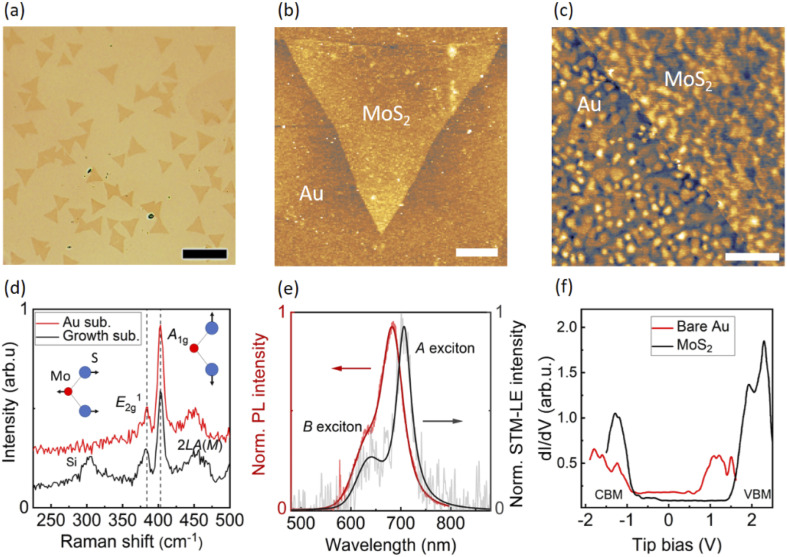
(a) Light microscopy image of typical CVD-synthesized monolayer MoS_2_ flakes transferred onto the evaporated gold substrate. Scale bar: 20 μm. (b) AFM topography of a monolayer MoS_2_ flake on the gold substrate. Scale bar: 2 μm. (c) STM topography obtained with a tip bias of −3 V and a tunneling current of 100 pA. Scale bar: 200 nm. (d) Raman spectra of monolayer MoS_2_ on growth substrate (SiO_2_/Si) and evaporated Au substrate. (e) PL and STM-LE spectra of monolayer MoS_2_ on evaporated Au substrate. Both Raman and PL are acquired by an *ex situ* setup with an excitation laser of 561 nm wavelength. STM-LE spectrum is obtained with a tip bias of −4.5 V, tunneling current of 30 nA and integration time of 3 min. (f) Differential conductance d*I*/d*V* spectra acquired on the bare gold surface and monolayer MoS_2_. The corresponding CBM and VBM are found at tip bias of −0.8 eV and 1.5 eV, respectively.

Fig. 1a shows an optical image of triangular-shaped monolayer MoS_2_ flakes wet-transferred onto the evaporated Au substrate. The substrate provides enough visual contrast between Au and MoS_2_ to unambiguously identify MoS_2_ flakes. Fig. 1b shows the surface topography of a monolayer MoS_2_ flake acquired by atomic force microscopy (AFM) in tapping mode. Fig. 1c displays a constant-current STM image of the same MoS_2_ flake with a smaller field of view. On the growth substrate (300 nm SiO_2_/Si) the monolayer MoS_2_ is confirmed by AFM height measurements, which show step heights of ∼1 nm for a single layer. The thickness of monolayer MoS_2_ is slightly higher than the reported thickness of monolayer MoS_2_ (∼0.8 nm).^14^ In tapping-mode AFM, the different tip-sample interactions between MoS_2_ and substrate add shifts to the phase, influencing the measured height. To confirm the single-layer of the MoS_2_ flakes, we performed Raman and PL experiments as will be discussed later. The increased height (∼2 nm) of MoS_2_ after wet-transfer onto the gold substrate (Fig. S1†) indicates the presence of water clusters underneath the MoS_2_ flake, which decouple the electronic interaction between MoS_2_ and Au substrate. Such a water spacer is frequently observed when transferring 2D materials in ambient conditions, especially when employing water-assisted transfer, as already found for WSe_2_ transferred onto Au^25^ and MoS_2_ on graphite.^31^ The surface corrugation of the underlying evaporated Au film translates into MoS_2_, as evidenced by the STM topography and confirmed by AFM. As a result, local variations in deformation and strain are to be expected in the monolayer MoS_2_.

Fig. 1d shows the *ex situ* Raman spectra of monolayer MoS_2_ as-synthesized on the growth substrate and after transfer onto the evaporated Au substrate, both measured in air at room temperature. On both substrates, the in-plane vibration mode *E*^1^_2g_ and the out-of-plane vibration mode *A*_1g_ of MoS_2_ are found at around 383 cm^−1^ and 403 cm^−1^, respectively. A difference of ∼20 cm^−1^ between the two modes is in good agreement with the typical characteristic reported for single-layer MoS_2_.^32^ The second order peak of longitudinal acoustic phonons at *M* point (2*LA*(*M*)) is also visible in Raman scattering.^32^ The absence of significant changes in the Raman spectrum after transferring MoS_2_ onto the Au substrate points to an electronic decoupling from the metallic surface by the water clusters.^25^Fig. 1e shows the *ex situ* PL and STM-LE spectra of the same monolayer MoS_2_ on evaporated Au substrate. The intense PL peak at ∼683 nm arises from the radiative recombination of *A* excitons,^2^ and the *A* peak in the STM-LE spectrum is found at ∼706 nm. The secondary STM-LE peak at a shorter wavelength (∼635 nm), blue-shifted by about 0.2 eV from the *A* peak, is assigned to the radiative decay of *B* excitons. This energy shift matches the valence band splitting energy induced by strong spin–orbit coupling in monolayer MoS_2_.^33,34^ This *B* exciton emission also contributes to the shoulder in the PL spectrum.

Owing to the different excitation methods, the PL spectrum presents the overall optical behavior of an area several hundred nanometers in diameter, while the STM-LE derives from local excitation on the nanometer scale. To better understand the spectral shift between PL and STM-LE spectra, we investigated PL of monolayer MoS_2_ flakes on different substrates (Fig. S2).† Monolayer MoS_2_ as-synthesized on the growth substrate and after transfer onto a fresh 300 nm SiO_2_/Si substrate show similar PL peaks. The slight broadening after transfer may be caused by the introduction of charges or defects during the transfer process.^35^ After transfer onto rough surfaces, *i.e.*, evaporated Au or indium tin oxide (ITO)-coated glass, the PL spectra of monolayer MoS_2_ exhibit red-shifts due to the strain introduced by the corrugated surface, as already reported previously.^36^ The larger PL red-shift of monolayer MoS_2_ on ITO compared to that of MoS_2_ on evaporated Au is in good accordance with previously reported results since ITO has a rougher surface.^36^ Consequently, we attribute the observed difference in peak wavelengths between the PL and STM-LE spectra to the local strain-induced exciton modulation resolved by STM-LE, which will be discussed in more detail below. The broadening of the PL on rough substrates may also result from the strain distribution in MoS_2_.

First, we turn to the characterization of electronic properties of monolayer MoS_2_ by scanning tunneling spectroscopy (STS) (see Fig. 1f). The reference d*I*/d*V* spectrum on bare evaporated Au shows the characteristic surface state at around 0.5 V tip bias. The d*I*/d*V* spectrum of MoS_2_ displays clear band edges: conduction band minimum (CBM) and valence band maximum (VBM) at around −0.8 V and 1.5 V, respectively, indicating the electronic band gap of the monolayer MoS_2_ to be 2.30 ± 0.09 eV (3 measurements). The corresponding *A* exciton binding energy (*i.e.*, the energy difference between the electronic band gap and the optical gap) is ≈ 0.5 eV resulting from an STM-LE *A* peak of ∼1.8 eV at the measured sample location. The electronic band gap of our monolayer MoS_2_ is closer to the band gap of ≈2.5 eV of suspended monolayer MoS_2_,^37^ in contrast to a band gap of 1.74 eV from epitaxial monolayer MoS_2_ grown directly on Au.^38^ For epitaxial MoS_2_ on Au, the interaction between the metal substrate and MoS_2_ leads to hybridization of the states and a lower band gap.^38^ The measured electronic band gap of our monolayer MoS_2_ on Au provides further evidence of decoupling water clusters in between.

We now compare the STM-LE of bare Au surface and monolayer MoS_2_. The light emission from the Au surface (Fig. S3†) is attributed to the radiative decay of gap plasmons, which are sensitive to the local geometry and dielectric properties.^39^ STM-LE spectra of monolayer MoS_2_ acquired at fixed sample location for both bias polarities are shown in Fig. S4.† For low tip bias voltage, there is only one emission peak from the plasmonic radiative decay. With increasing voltage, a second peak appears at a shorter wavelength, corresponding to the excitonic radiative decay of MoS_2_, which barely shifts when tip bias is high. Despite its presence, the plasmonic background does not hinder the study of the optical properties of monolayer MoS_2_, as the plasmonic background is weaker than the excitonic emission at high tip bias (>3 V).

The photon emission quantum efficiency of MoS_2_ is obtained by simultaneously scanning the sample surface with STM tip and recording the photon number with the photon counter. With a tip bias of −3.25 V, the averaged photon count is 350 s^−1^nA^−1^ (Fig. S5†). Accounting for the geometric collection efficiency of the lens system and the photon counter's detection efficiency, the quantum efficiency (QE) of STM-LE of monolayer MoS_2_ is estimated to be 3.7 × 10^−6^ photons per electron, similar to the value reported for MoSe_2_/Au (∼4 × 10^−6^ photons per electron).^24^

### Tunneling current-induced exciton emission

**Fig. 2:**
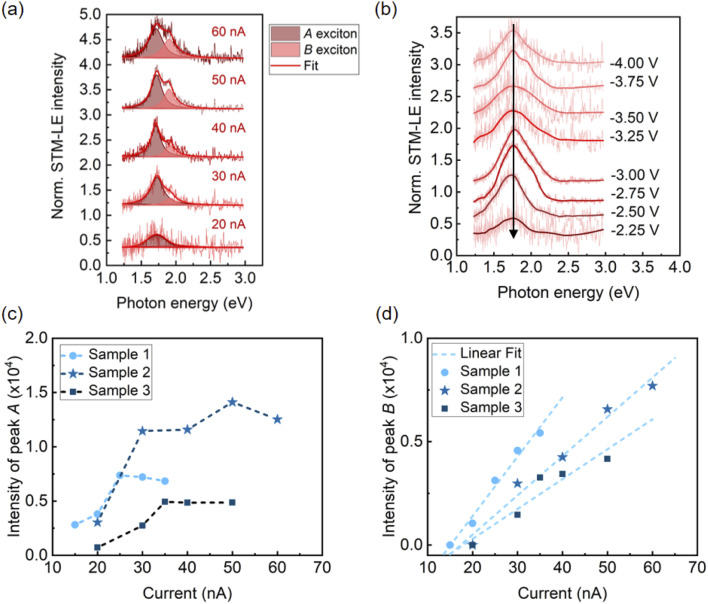
The influences of tunneling current and tip bias on STM-LE spectra of monolayer MoS_2_. (a) Normalized STM-LE spectra acquired at a fixed sample location with different tunneling currents. Tip bias: −4 V. The spectra are well fitted with two Lorentzian peaks associated with *A* exciton (dark pink) and *B* exciton (light pink). (b) Normalized STM-LE spectra at a fixed sample location of MoS_2_ with varying tip biases. Tunneling current: 30 nA. (c) and (d) show the current-dependent intensities for *A* and *B* excitons, respectively. The intensities are extracted from fitted spectra obtained at different locations on the same MoS_2_ flake. The data in (d) is fitted linearly.

Exciton emission of TMDCs has been reported to depend on current. Examples include multiple-exciton–exciton interactions in a MoS_2_ diode^30^ and neutral exciton and trion emission controlled by tunneling current.^25^ Using STM-LE, we studied the influence of local tunneling current and tip bias on exciton emission from monolayer MoS_2_. The STM-LE spectra acquired at a fixed sample location with a constant tip bias are shown in Fig. 2a. The spectra indicate a systematic change of exciton emission with different current settings. When the tunneling current is around 20 nA, the spectrum only shows one emission peak, corresponding to the *A* exciton recombination. The *B* peak appears for higher tunneling currents. For the current and bias ranges probed in our measurements, tunneling current only influences the exciton emission intensity but not the exciton energy, as the spectral peaks do not shift with different current settings. Fig. 2b further shows that the tip bias does not affect the exciton energy.

Understanding the exciton dynamics of monolayer MoS_2_ is essential for device development. The current-dependent emission intensities of *A* and *B* excitons are presented in Fig. 2c and d. The STM-LE intensity is related to the exciton lifetime (*τ*_ex_), which depends on the radiative and nonradiative exciton decay through:^40^
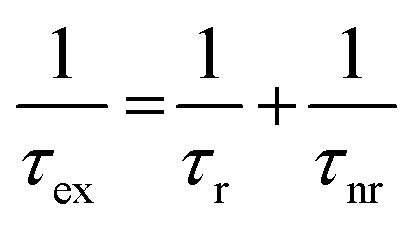
where *τ*_r_ and *τ*_nr_ are the radiative and non-radiative decay times, respectively. The STM-LE intensity is proportional to *τ*_ex_/*τ*_r_.^40^ Radiative recombination rate is an intrinsic property, which shows little change from sample to sample for the same material at a fixed temperature in similar environment.^41^ The negligible changes of the peak positions and full-width-half-maximums for both *A* and *B* exciton emissions (Fig. S6†) indicate no heating effect on the sample through the measurements. Thus, the intensity difference among different sample locations for a given tunneling current (slopes in Fig. 2d) is mainly caused by variations in the local environment, for instance, local defect density^42^ or substrate doping.^43^ This is applicable for both *A* and *B* excitons.

In Fig. 2, the *B* peak intensity shows a linear dependence on the tunneling current in the range of 20 nA–60 nA. In contrast, *A* peak intensity saturates under high tunneling currents. This saturation of *A* peak intensity can be explained by the non-linear process of exciton–exciton annihilation (EEA), which has been widely observed in monolayer TMDCs in photoluminescence measurements.^40,44^ For high exciton density, EEA opens an additional path for non-radiative exciton decay. In steady state, the rate of change in the population *N* of excitons excited by tunneling current injection can be described as:^44^
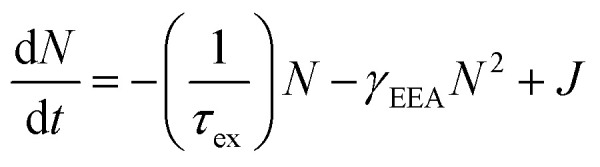
where, *γ*_EEA_ is the EEA rate, and *J* is the injection-current-associated excitation. The STM-LE intensity is proportional to the exciton population *N*. When *N* is low, the exciton decay is determined by the linear radiative and non-radiative processes and the light intensity shows a linear dependence on the excitation current. When the exciton population is large (
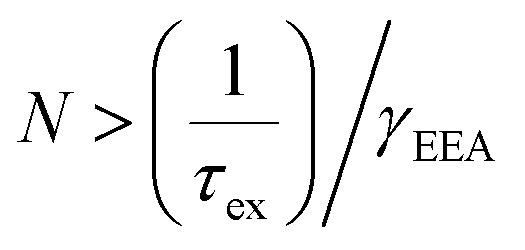
), EEA becomes the major exciton decay process, causing the current-dependent STM-LE intensity to deviate from the linear trend.

In our measurements, the required injection current for *A* exciton to reach the EEA-dominated population regime is lower than that for *B* exciton. This difference can be explained by the different exciton lifetimes and EEA rates between *A* and *B* excitons. Particularly, the rapid relaxation (∼ps) from *B* exciton to *A* exciton reduces the *B* exciton population and simultaneously increases the *A* exciton population.^45^ This rapid relaxation also explains the higher intensity of the *A* peak compared to the weak *B* peak at low currents before reaching the EEA-dominated regime. More quantitative analysis of exciton dynamics would require ultrafast time-resolved spectroscopy, which is beyond the scope of our setup. Knowing the EEA rate helps control the excitation injection to maintain an optimal light emission efficiency of TMDCs.

### Strain-induced exciton emission

**Fig. 3:**
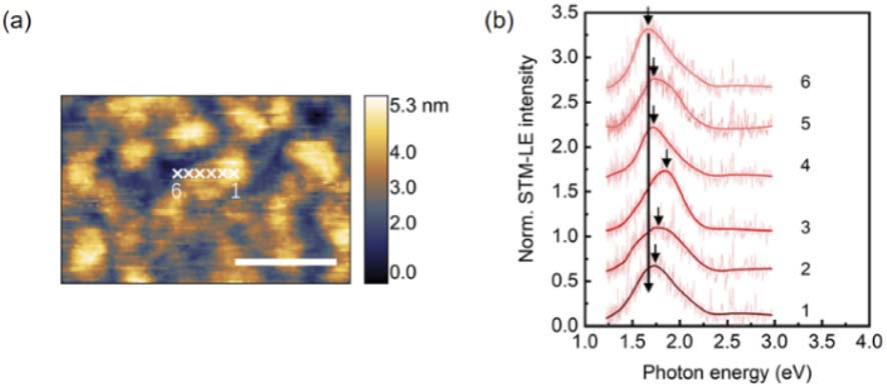
The influence of substrate topography on STM-LE spectra of monolayer MoS_2_. (a) and (b) show STM topography and STM-LE spectra, respectively. The spectra are obtained at six sample locations separated by 10 nm as marked in (a). STM parameters: −3 V tip bias, and 100 pA tunneling current. Scale bar: 100 nm. STM-LE parameters: −4 V tip bias, 30 nA tunneling current, and 3 min integration time.

Monolayer MoS_2_ exhibits high mechanical flexibility.^46^ Placed on an evaporated Au substrate, van der Waals interactions make the as-grown planar monolayer MoS_2_ conform to the local surface corrugation, which causes locally varying levels of strain. No wrinkles or bubbles are observed after transfer by optical microscopy and AFM. We assume the strain is mainly introduced by the surface roughness of the Au substrate. Strain tunes the electronic band gap but not the binding energy of excitons.^17,18,47^ Thus, we observe significant peak shifts of exciton emission as shown in Fig. 3, where STM-LE spectra are acquired at sample locations separated by 10 nm.

Assuming that shear strain components are negligible, biaxial strain *ε*_b_ takes the simple form of *ε*_*xx*_ + *ε*_*yy*_, where *ε*_*xx*_ and *ε*_*yy*_ are the strain components in the *x* and *y* directions.^48,49^ To derive the sum of these two strain components from local surface topography, we compare the actual surface area of MoS_2_ and its projected area, as shown in Fig. S7.† To this end, a Gaussian filter is applied to the local surface topography to generate smooth surfaces. Then, to first order approximation, the tensile biaxial strain is obtained by:
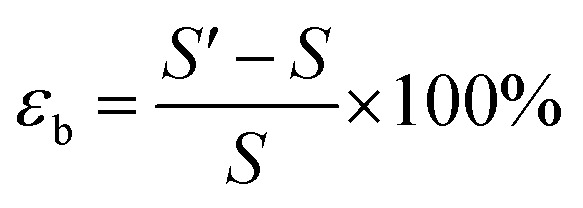
where *S*′ is the actual surface area and *S* is the projected area.

#### Low tunneling current

**Fig. 4:**
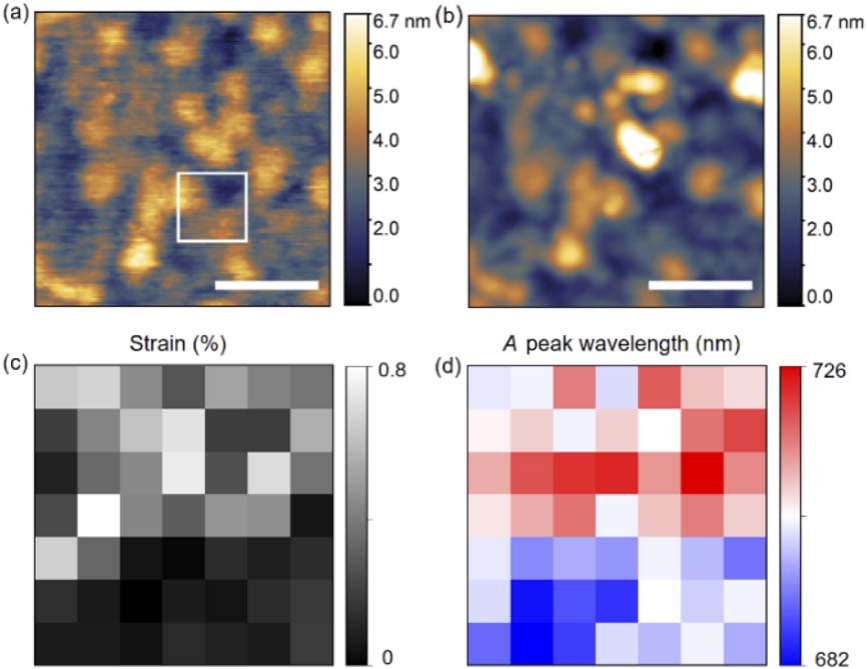
The influence of strain on STM-LE spectra of MoS_2_. (a) STM image of monolayer MoS_2_ acquired with a tip bias of −3 V and a tunneling current of 100 pA. Scale bar: 100 nm. (b) AFM image of MoS_2_ at the same sample region as (a). Scale bar: 100 nm. (c) Strain map and (d) corresponding *A* peak wavelength map acquired at the region indicated by the white box in (a). The pixel size is 10 × 10 nm^2^. The exciton energies are determined by fitting the spectra with Lorentzian profiles. STM-LE parameters: −4 V tip bias, 30 nA tunneling current, and 3 min integration time.

To identify the bandgap modulation by strain, we performed STM-LE on monolayer MoS_2_ with low tunneling currents where the *A* peak dominates. Fig. 4a shows an STM image of monolayer MoS_2_ on Au substrate, acquired with constant tunneling current and tip bias. The AFM image of the same area, Fig. 4b, shows identical surface structures. We conclude that the height information in STM image is determined by the surface topography of the monolayer MoS_2_. The area marked by the white box in Fig. 4a, which includes surfaces with different local curvatures, is evaluated by STM-LE. Fig. 4c presents the strain distribution and Fig. 4d the corresponding STM-LE peak wavelength of *A* excitons with pixel size of 10 × 10 nm^2^. The spectral shifts correlate with the local strain of the monolayer MoS_2_. A 44 nm red shift is observed between the locations with the highest and lowest strains.

**Fig. 5:**
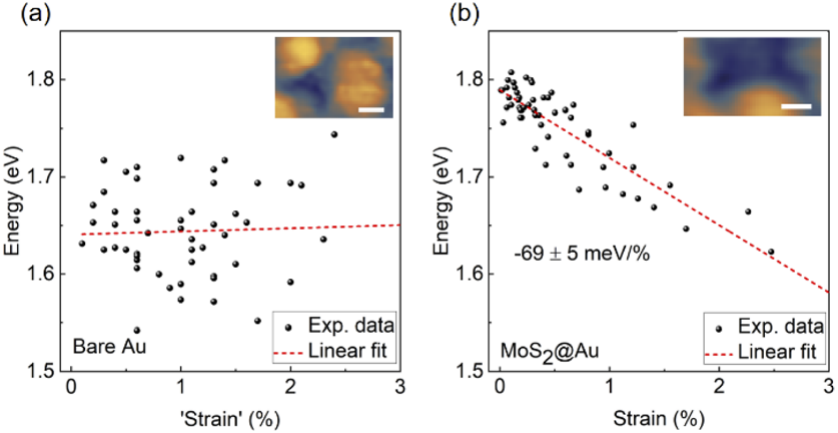
(a) Peak energy of STM-LE on the bare gold surface with different local curvature. (b) *A* exciton energy of MoS_2_ with different local strains induced by curvature. Inserts show the corresponding STM images. Scale bar: 20 nm. The pixel size for each data point is 10 × 10 nm^2^. The data points are fitted linearly with Pearson correlation coefficients of 0.06 and −0.87 for (a) and (b), respectively. The modulation rate of strain on *A* exciton is estimated as −69 ± 5 meV/%.

Fig. 5 shows the variation of measured peak energies with local curvature induced strain. Fig. 5a displays the plasmonic peak energy of the bare Au substrate *versus* a virtual ‘strain’ value, calculated by the same method described above for MoS_2_. The absence of a clear trend in energy variation over a range of local curvatures is evident. We attribute the comparatively wide distribution of peak energies for identical virtual ‘strain’ values to local variations in the plasmonic tip–sample cavity. In contrast, *A* exciton peak energy of MoS_2_, shown in Fig. 5b, exhibits a clear linear modulation rate of −69 ± 5 meV/% with a Pearson correlation coefficient of −0.87. This modulation rate is in good agreement with the result obtained by diffraction-limited photoluminescence of CVD monolayer MoS_2_ upon biaxial strain (a value of −76 ± 10 meV/%).^48^ Considering the absence of a trend on the bare Au substrate and the electronic decoupling of monolayer MoS_2_ from the Au substrate by a thin water spacer, the observed linear modulation of *A* exciton energy can be assigned to the local strain variations in MoS_2_, induced by the corrugated surface. Moreover, spectra acquired on other monolayer MoS_2_ flakes wet-transferred from the same CVD-synthesis have closely matching values for the strain modulation rates (Fig. S8†).

#### High tunneling current

**Fig. 6:**
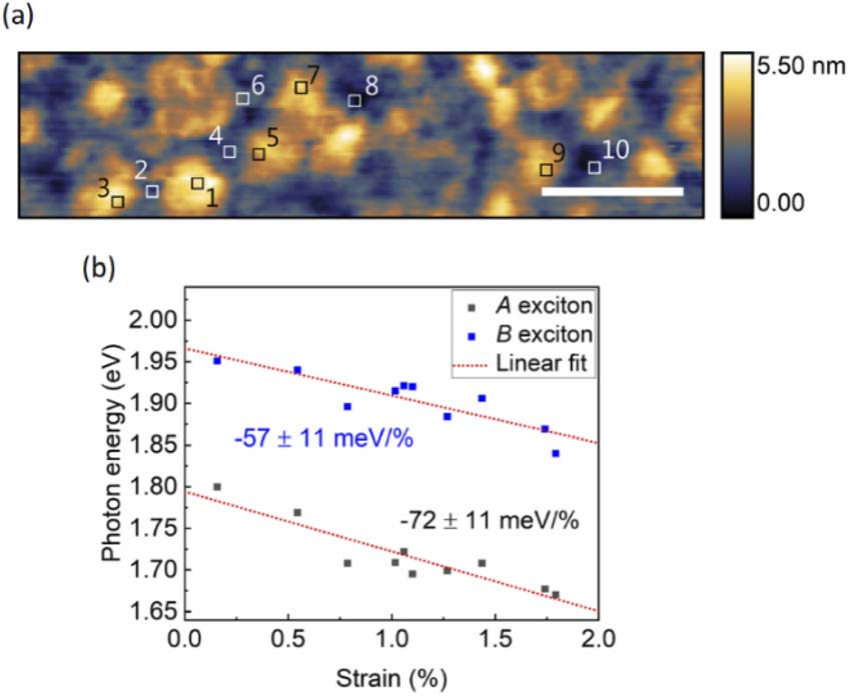
Strain-modulated *A* and *B* excitonic luminescence. (a) STM image of monolayer MoS_2_ on Au surface. Tip bias: −3 V, tunneling current: 100 pA, scale bar: 100 nm. (b) *A* and *B* exciton energies as functions of strain, acquired at the locations indicated by the boxes in (a). Tip bias: −4 V, tunneling current: 50 nA, integration time: 3 min.

We further investigated the influence of strain on the band structure of monolayer MoS_2_ under high tunneling currents, where both *A* and *B* peaks are visible. In addition to modifying the bandgap, the strain could also affect band splitting. In Fig. 6, STM-LE spectra are recorded on monolayer MoS_2_ at locations with different surface curvatures (*i.e.*, strain). The area for each location is 10 × 10 nm^2^. The local strain is calculated by the same method as described above. Both *A* and *B* exciton energies show linear dependences on strain, like the results obtained for low tunneling currents. The *B* exciton energy decreases with strain at a rate of −57 ± 11 meV/%, which is different from *A* exciton energy. This deviation between *A* and *B* exciton energy shifts provides evidence that the valence band splitting changes with strain, which is consistent with theoretical calculations.^47,50^

## Discussion

Two possible excitation mechanisms can be involved in STM-LE: (i) diodelike excitation through electron and hole injections^25,51,52^ and (ii) resonance energy transfer by virtual photon coupling.^23^ The diodelike excitation mechanism generally requires a luminescence onset electron energy (*i.e.* tip bias) higher than the ‘free particle’ electronic bandgap energy of monolayer MoS_2_. In the resonance energy transfer mechanism, the onset of luminescence occurs at an electron energy surpassing the optical gap energy. In our work, the onset of luminescence of MoS_2_ (Fig. S4†) occurs at a negative tip bias close to the optical gap of monolayer MoS_2_, indicating that the luminescence is excited by virtual photon coupling. Due to the limited sensitivity of our spectrometer, all STM-LE spectra were acquired with a tip bias higher than the electronic bandgap of monolayer MoS_2_, where both excitation processes may be present.

For the range of tip bias and current applied in our experiments, STM-LE spectra show no systematic peak shifts at fixed locations for varying tip bias or tunneling current. This holds both for plasmonic emission from the bare Au substrate and excitonic emission from MoS_2_. Hence, the tunneling gap or the electric field have little influence on the exciton energies of MoS_2_. The presence of water clusters between Au substrate and MoS_2_ effectively decouples the two materials electronically, allowing one to directly probe the excitonic emission of MoS_2_. The spectral shifts in excitonic luminescence in monolayer MoS_2_ are directly related to alterations of the electronic bandgap induced by strain.

In strain-engineered 2D materials, thermal scanning probe lithography has recently achieved a strain pattern with 20 nm resolution.^22^ It is challenging to adequately resolve such a fine pattern by far-field optical excitation methods. Local excitation as in STM-LE offers a powerful alternative. However, excitons may diffuse up to 2 μm away from the location of excitation before radiative decay occurs at room temperature.^23^ By locally modifying the electronic band gap of 2D materials, strain also can introduce funnel centers towards which excitons drift before recombination.^21^ Thus, resolving local strain *via* spectral signatures requires consideration of the measuring methods and the exciton dynamics. In our experiments, taking advantage of STM-LE, local strain changes in monolayer MoS_2_ can be distinguished on a length scale of 10 nm, as evidenced in Fig. 3 and 4.

## Conclusion

STM-LE is a powerful technique to probe excitons in confined semiconductors with nanometer lateral resolution. In this work, we present a study of the excitonic luminescence of monolayer MoS_2_ on an evaporated gold thin film substrate, locally excited by an STM tip. Due to the water spacer between substrate and MoS_2_, electronic coupling between substrate and MoS_2_ is negligible, allowing one to investigate the excitonic emission of monolayer MoS_2_ despite the presence of a plasmonic background. The luminescent spectra from monolayer MoS_2_ are attributed to the radiative recombination of *A* and *B* excitons. Both *A* and *B* excitonic peaks show energy shifts due to the local strain introduced by the corrugated substrate. Additionally, the emission intensities of *A* and *B* excitons depend on tunneling current. Thus, by tuning the tunneling current, the luminescence spectra can be adapted to different investigations. For instance, the local strain distribution of monolayer MoS_2_ can be probed with a low tunneling current through analyzing the *A* exciton energy, which avoids long-term heating. Exciton energies and dynamics (in particular, exciton lifetimes) can be explored with high tunneling currents, where both *A* and *B* exciton emissions are detectable. In addition to investigating the optoelectronic properties of 2D materials, STM-LE also enables one to perform local analyses of strain or material deformation in piezoelectrical^53,54^ and piezo-resistive devices.^55^

## Experimental

### Materials

Sulfur (S) powder (99.98%, Sigma-Aldrich, CAS: 7704-34-9) and sodium molybdate (Na_2_MoO_4_) powder (≥98%, Sigma-Aldrich, CAS: 7631-95-0) were used as delivered and not purified further. The MoS_2_ flakes were grown on a Si(100) n-type substrate, covered with a 300 nm thick SiO_2_ layer, and synthesized in a 1-inch single heating zone tube furnace (Lindberg/Blue M). Quality and thickness of flakes were investigated by optical microscopy and atomic force microscopy.

### Methods

#### CVD synthesis

The SiO_2_/Si substrate was cleaned in an ultrasonic bath with acetone, isopropanol (IPA), and de-ionized (DI) water for 15 to 20 minutes. Prior to placing the molybdenum source directly onto the cleaned substrate by spin-coating an aqueous Na_2_MoO_4_ solution, the substrate was treated with O_2_ plasma to increase the hydrophilicity. The substrate was positioned at the center of the furnace, and 2 g of sulfur were placed in a crucible at the entrance of the furnace in the upstream heating zone. After the substrate and sulfur were loaded, the tube was flushed with 500 and 100 sccm N_2_ before the start of the heating process and during the synthesis, respectively. The temperature was gradually increased to 750 °C within 20 minutes and held for 15 minutes before cooling down. To accelerate the cooling, the furnace was opened partly at 650 °C and completely at 570 °C.

#### Transfer

The flakes grown on 300 nm SiO_2_/Si were transferred onto different substrates: evaporated gold substrate (100 nm Au on SiO_2_), 300 nm SiO_2_/Si, 120–160 nm ITO coated glass (Sigma-Aldrich, CAS: 50926-11-9), using a modified polymethyl methacrylate (PMMA) mediated transfer method.^56^ To this end, the growth substrate was covered with PMMA (950K) by spin-coating. After curing overnight, the edges of the substrate were cut to increase the penetration of liquid and to cut off the flakes grown under the influence of the substrate edge. To peel off the PMMA layer, the substrate was floated on a 2 M KOH solution. Afterwards, the PMMA layer was washed three times with DI water before being transferred to a fresh substrate and dried overnight. To dissolve the PMMA layer, the substrate was immersed in acetone, IPA, and DI water for 1 minute per solvent for three cycles.

### STM-LE setup

STM-LE experiments were conducted at room temperature in high vacuum (10^−7^ mbar), using a custom-built STM instrument. An aspheric lens (Thorlabs A110-B, NA 0.40) mounted at an incident angle of 60° from the sample normal collects the emitted light. In the case of isotropic radiation, the hemisphere photon collection efficiency is about 8.3%. However, the light emission pattern is modified by the tip-sample junction and the orientations of the luminescent exciton/dipole.^57,58^ Thus, the collection efficiency could be higher due to the angle-dependent emission pattern. In our experiments, we estimate the final detection efficiency of the optical system by only considering isotropic radiation. An optical fiber (Schaefter + Kirchhoff V-KF40-2x-MMC-VIS/NIR-105-NA022) guides the light out of the vacuum chamber to detectors. The STM-LE is either recorded by a photon counter (Hamamatsu C1300-1) or a spectrograph (Princeton Instrument SP2156i, with a 150 lines/mm grating) and a cooled CCD camera (PCO 2000) or a cooled EMCCD (Andor Newton 970P). The differential conductance d*I*/d*V* is measured by STS with a lock-in amplifier (modulation voltage: 50 mV, and frequency: 470 Hz). All STM measurements are acquired with platinum/iridium (90 : 10) tips prepared by electrochemical etching in CaCl_2_ solution. The STM tips have radii of 50∼100 nm supporting lateral resolution in the few nm range. Additionally, surface topography is acquired by an atomic force microscope (AFM) (Oxford Instruments, Cypher).

### Raman and PL setup

Both Raman and PL measurements were performed in air at ambient condition by a NT-MDT Raman system equipped with a 100× objective (NA = 0.8), using an excitation laser of 561 nm. Gratings: 150 lines/mm (PL measurement) and 600 lines/mm (Raman measurement) were used.

## Conflicts of interest

There are no conflicts to declare.

## Supplementary Material

RA-012-D2RA05123K-s001
