# Supplementary material for: Local strain and tunneling current modulate excitonic luminescence in MoS2 monolayers
Source: RSC Adv. 2022 Sep 1;12(38):24922–9. doi: 10.1039/d2ra05123k (PMC9434384; doi:10.1039/d2ra05123k)
Supplement: RA-012-D2RA05123K-s001 [file RA-012-D2RA05123K-s001.pdf]

Electronic Supplementary Material (ESI) for RSC Advances.
This journal is © The Royal Society of Chemistry 2022

Supporting information

Local strain and tunneling current modulate excitonic luminescence in MoS_2 monolayers

Yalan Ma*[1], Romana Alice Kalt[1], and Andreas Stemmer*[1]

Address: [1]Nanotechnology Group, ETH Zürich, Säumerstrasse 4, 8803, Rüschlikon, Switzerland

Email: Yalan Ma* - mayala@student.ethz.ch, Andreas Stemmer* - astemmer@ethz.ch

* Corresponding author

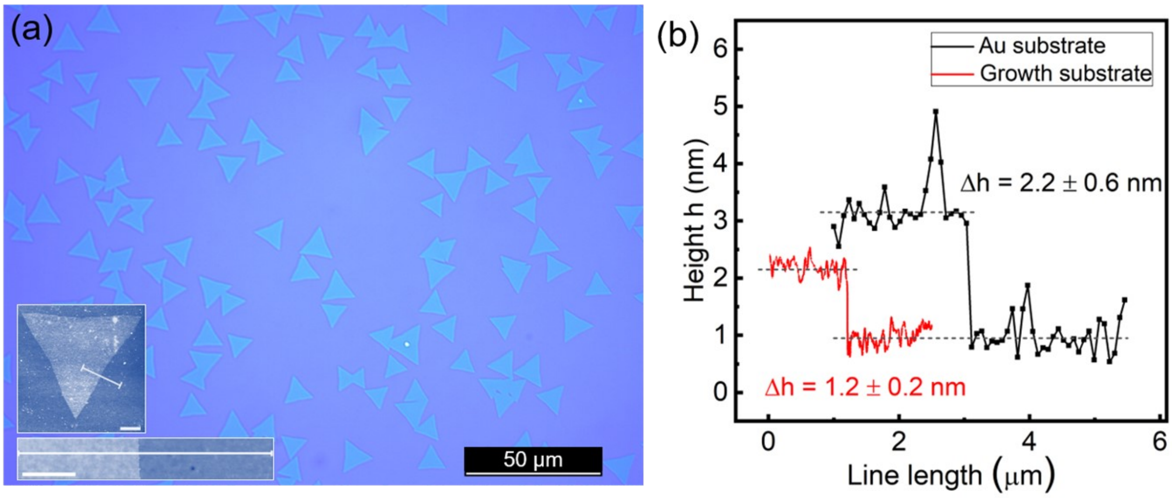

Figure S1: (a) Optical image of monolayer MoS_2 as synthesized on the growth substrate (300 nm SiO_2/Si). The two insets show the AFM images of monolayer MoS_2 on the growth substrate (rectangular figure, scale bar: 500 nm) and after wet-transfer onto the evaporated Au surface (square figure, scale bar: 2 μm). (b) AFM height profiles of monolayer MoS_2 on the growth substrate and evaporated gold as indicated in (a). Step heights of MoS_2 on the growth substrate and the gold substrate are 1.2 ± 0.2 nm and 2.2 ± 0.6 nm, respectively. The standard deviation represents the surface roughness.

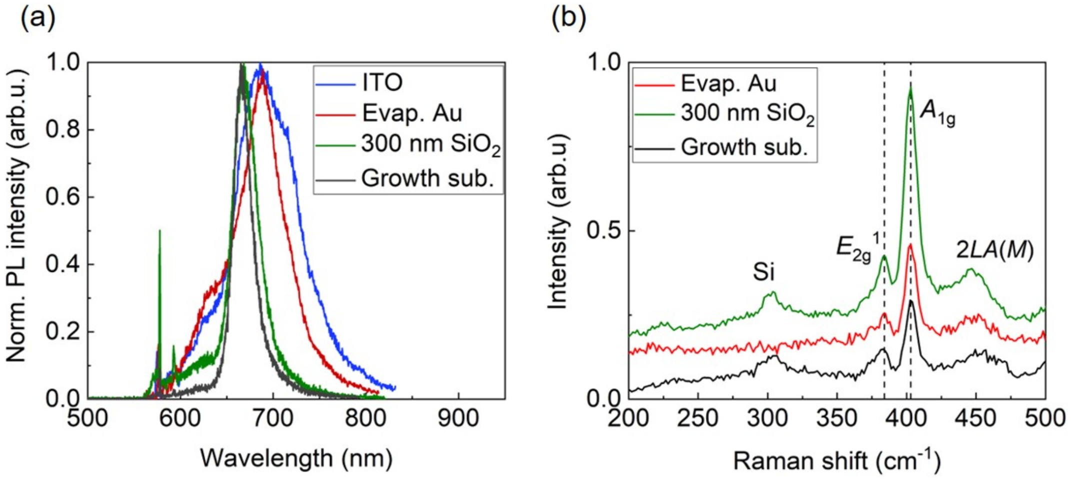

Figure S2: (a) Normalized PL spectra and (b) Raman spectra of monolayer MoS_2 on different substrates: as-synthesized on the growth substrate, after transfer onto ITO, evaporated Au, and 300nm SiO_2/Si substrates.

The in-plane vibrational mode E_{2g}^{1} and the out-of-plane vibrational mode A_{1g} of monolayer MoS_2 have been reported to be modulated by strain [1, 2]. Michail *et al.* [2] has presented shift rates of -3.1 cm^{-1}/% for E_{2g}^{1} mode, -1.7 cm^{-1}/% for A_{1g} mode and -76 meV/% for *A* excitonic PL peak (close to our value: -69 meV/%). In our monolayer MoS_2 transferred onto evaporate Au surface, the introduced strain is estimated as ~0.58% from a redshift of ~40 meV in PL peaks. If we use the shift rates reported by Michail *et al.* [2], a shift of -1.8 cm^{-1} for E_{2g}^{1} peak and -1.0 cm^{-1} for A_{1g} peak would show in the Raman signal of monolayer MoS_2 on Au. However, due to the limited resolution of our Raman setup (~1.5 cm^{-1}/pixel) and the low signal-to-noise ratio, no significant peak shift is observed in the Raman signal.

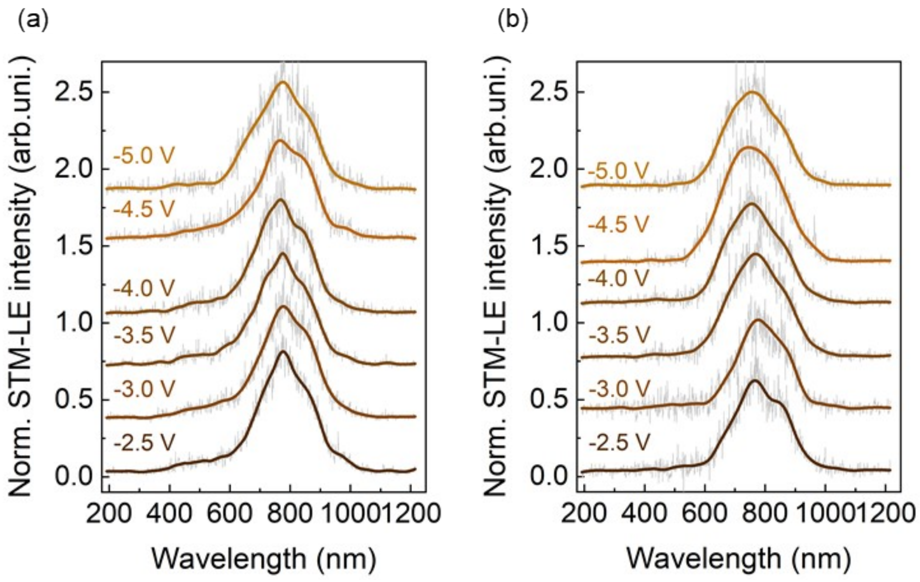

Figure S3: (a) and (b) show the spectra with variable tip bias, obtained from bare Au surface at two different locations. The peak wavelengths show no dependence on the tip bias.

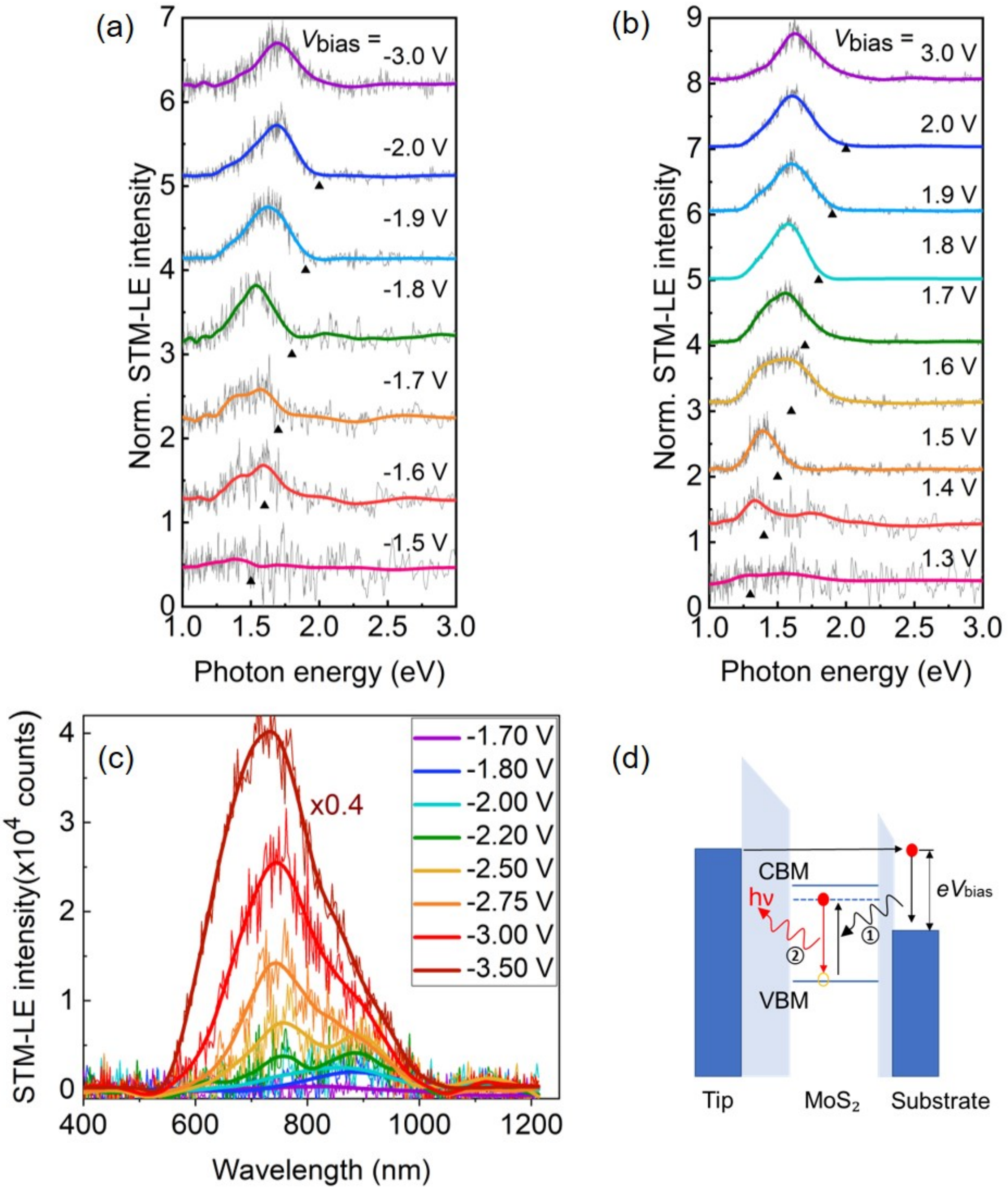

Figure S4: (a) and (b) Normalized STM-LE spectra of monolayer MoS_2 as a function of tip bias. Gray lines are normalized raw data. Colored lines are smoothed curves. The black triangles mark the position of expected quantum cutoff (i.e. photon energy equals maximum tunneling electron energy). (c) shows the STM-LE spectra of monolayer MoS_2 at different tip biases. Tunneling current: 50 nA, and integration time: 120 s. (d) displays a schematic on resonant energy transfer mechanism for negative tip bias. When electron energy exceeds optical gap of monolayer MoS_2, the tunneling electrons ① transfer their energy to MoS_2 via virtual photon coupling, generating excitons in MoS_2. The excitons then decay radiatively and emit photons ②.

In Figure S4 (a), the STM-induced luminescence begins at bias voltage of ~-1.6 V which matches more closely the optical band gap (~1.65 eV) than the electronic band gap. Thus, excitons are created via resonant energy transfer mechanism [3]. To explain the over-bias light emission (photon energy exceeds the quantum cutoff) in Figure S4 (a) and (b), additional experiments are needed to elucidate the mechanisms behind.

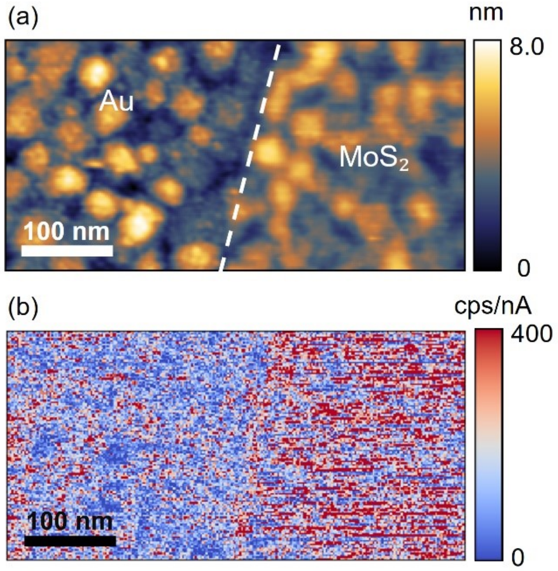

Figure S5: (a) STM topography of MoS_2 on evaporated Au surface. Tip bias: -3.25 V. Tunneling current: 1 nA. (b) Corresponding photon count map simultaneously acquired by the photon counter when scanning the area in (a) by STM tip.

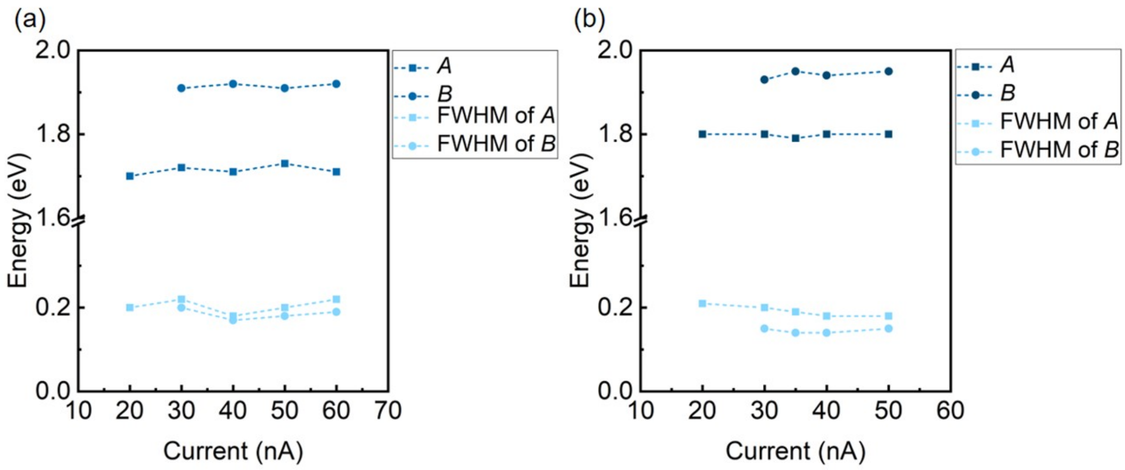

Figure S6: (a) and (b) show the peak energies and the full-width-half-maximum (FWHM) of *A* and *B* exciton emission peaks for sample 2 and 3 in Figure 2, respectively.

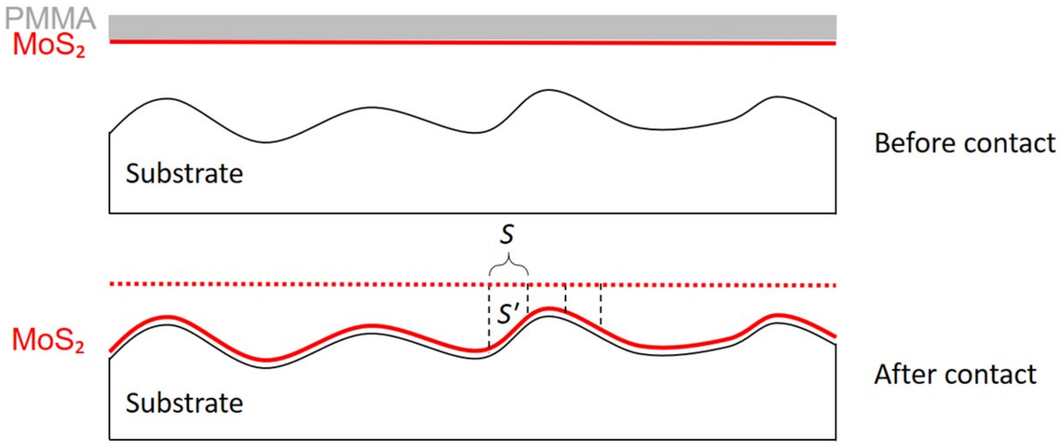

Assuming:

$$S = xy$$

$$S' = (x+\Delta x)(y+\Delta y)$$

Then:

$$\varepsilon_b = \frac{S'-S}{S} = \frac{\Delta x}{x} + \frac{\Delta y}{y} + \frac{\Delta x \Delta y}{xy} \approx \frac{\Delta x}{x} + \frac{\Delta y}{y} = \varepsilon_{xx} + \varepsilon_{yy}$$

Figure S7: Schematic of MoS_2/Au interface before and after contact. Substrate corrugation is exaggerated with respect to the thickness of MoS_2. S' and S are the surface areas of MoS_2 with and without deformation, respectively. By ignoring the higher order term, the biaxial strain ($= \varepsilon_{xx} + \varepsilon_{yy}$ [2,4]) is then estimated by comparing S and S'.

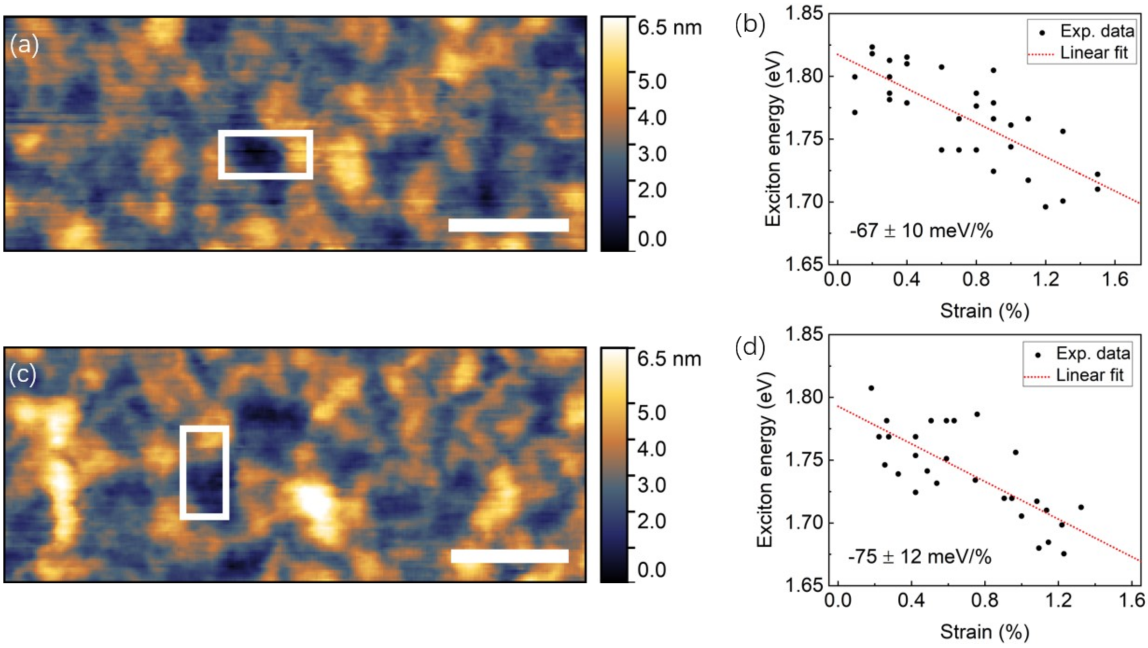

Figure S8: Strain measurements on different monolayer MoS_2 flakes transferred from the same CVD-synthesis onto the same evaporated Au substrate. (a) and (c) show STM topography of different monolayer MoS_2 flakes on evaporated Au substrate. Tip bias: -3 V. Tunneling current: 100 pA. Scale bar: 100 nm. (b) and (d) show *A* exciton energy as a function of strain obtained in the region indicated with white boxes in (a) and (c), respectively. Pixel size: 10×10 nm^2.

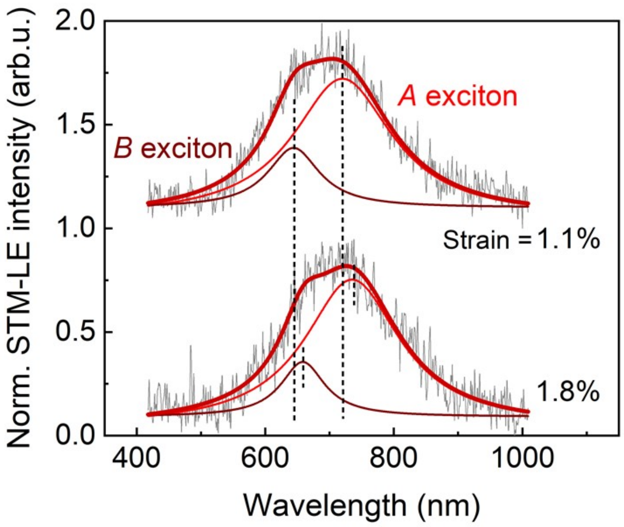

Figure S9: Normalized STM-LE spectra of monolayer MoS_2 with different strain values. The positions of the *A* and *B* excitonic peaks are marked by dash lines from Lorentzian fitting. Tip bias: -4 V, tunneling current: 50 nA, integration time: 3 min.

Reference

1. Li, H.; Contryman, A. W.; Qian, X.; Ardakani, S. M.; Gong, Y.; Wang, X.; Weisse, J. M.; Lee, C. H.; Zhao, J.; Ajayan, P. M.; Li, J.; Manoharan, H. C.; Zheng, X. Nature Communications 2015, 6, 1-7.

2. Michail, A., Anestopoulos, D., Delikoukos, N., Parthenios, J., Grammatikopoulos, S., Tsirkas, S. A., Lathiotakis, N. N., Frank, O., Filintoglou, K., Papagelis, K. 2D Mater., 2021, 8, 015023.

3. Pommier, D.; Bretel, R.; López, L. E. P.; Fabre, F.; Mayne, A.; Boer-Duchemin, E.; Dujardin, G.; Schull, G.; Berciaud, S.; Le Moal, E. *Phys. Rev. Lett.* **2019,** *123,* 027402.

4. Lăzărescu, L., COMŞA, D. S., Nicodim, I., Ciobanu, I., & Banabic, D. *Transactions of Nonferrous Metals Society of China,* **2012,** *22,* s275-s279.
